# Mental Health, Social and Emotional Well-Being, and Perceived Burdens of University Students During COVID-19 Pandemic Lockdown in Germany

**DOI:** 10.3389/fpsyt.2021.643957

**Published:** 2021-04-06

**Authors:** Elisabeth Kohls, Sabrina Baldofski, Raiko Moeller, Sarah-Lena Klemm, Christine Rummel-Kluge

**Affiliations:** Department of Psychiatry and Psychotherapy, Medical Faculty, Leipzig University, Leipzig, Germany

**Keywords:** mental health, COVID-19, COVID-19 lockdown, University students, depressive symptoms, burden, perceived stress, pandemic (COVID-19)

## Abstract

**Background:** The COVID-19 pandemic has been affecting everyone's daily life in unknown measures since its outbreak. Nearly all Universities around the globe were affected. Further, young people and University students in particular, are known to be vulnerable for developing mental disorders. This study aims to examine the mental health social and emotional well-being and perceived burdens of University students during COVID-19 pandemic lockdown in Germany.

**Materials and Methods:** This cross-sectional and anonymous online survey among University students assessed mental health status with standardized measures (depressive symptoms, alcohol and drug consumption, and eating disorder symptoms), attitudes toward the COVID-19 pandemic and perceived burdens, and social and emotional aspects of the pandemic (social support, perceived stress, loneliness, and self-efficacy).

**Results:** In total, *N* = 3,382 German University students participated. Nearly half of the students (49%) reported that they are worried or very much worried about the COVID-19 pandemic. The majority supports the governmental lockdown measures (85%). A Patient Health Questionnaire-9 (PHQ-9) sum score of 10 or above, indicating clinically relevant depressive symptoms, was reported by 37% (*n* = 1,249). The PHQ-9 sum score was on average 8.66 (*SD* = 5.46). Suicidal thoughts were indicated by 14.5% of the participants. Levels of depressive symptoms differed significantly for the different self-rated income changes during the pandemic (increase, decrease, no change in income). Further, levels of depressive symptoms and suicidal ideation differed significantly for students from different faculties. Multiple regression analyses revealed that not being a parent, having no indirect social contact one or two times a week, higher perceived stress, higher experienced loneliness, lower social support, and lower self-efficacy significantly predicted higher scores of depressive symptoms, also higher hazardous alcohol use, and higher levels of eating disorder symptoms. Other aspects of lifestyle such as social and cultural activities, dating, and hobbies were reported to be negatively affected during the pandemic.

**Conclusion:** The present study implies that University students are vulnerable and due to elevated depressive symptoms at risk, being hit hard by the pandemic, but are in general coping adaptively. Low-threshold online interventions promoting help-seeking and also targeting various mental health conditions might bridge the gap the COVID-19 pandemic opened up recently.

## Introduction

It is widely known that 75% of all severe mental illnesses occur before the age of 24 (1). Students in particular seem to have an increased risk of developing mental health problems [e.g., (2–4)]. Academic pressure (5), financial worries (6), and fear of the future (7) are just some of the stressors in everyday student life that can promote the occurrence of a mental illness.

Due to the outbreak of the COVID-19 virus (8), which occurred in China for the first time at the end of 2019 and which has now expanded into a global pandemic (9), another stress factor for the mental health of students was added. Students around the globe are affected by the pandemic in various areas of their life, but especially due to closing of campuses and face-to-face (f2f) teaching being replaced by online formats, reduction or elimination of social contacts and cultural activities.

Over the last months, a number of empirical studies in various countries has investigated the influence of the pandemic on students, attempting to answer the “timely call for action to further research on students' mental health” (10). A longitudinal study showed that Chinese students between the ages of 12 and 21 reported stronger signs of psychological stress than other age groups (11). A cross-sectional study from the UK replicated these findings; increased depression and anxiety were associated with being young, but also with being female, living alone, and being in a medical risk group (12). Another Chinese study in college students revealed that 24.9% of the 7,143 participants reported fear of the COVID-19 virus (13). The reported COVID-19 related stressors, which were associated with higher levels of anxiety in students, were a. financial turmoil, b. changes in daily life, and c. academic delays (12). A survey in Turkish students showed that 38% of respondents were worried about COVID-19 (14). Further, Greek students reported increased levels of anxiety (42.5%), depression (74.3%), and suicidal thoughts [63.3%; (15)].

Our present survey aims to provide a comprehensive overview about how the pandemic is affecting University students. University students are in general at risk for developing common mental health disorders around the globe (16–19) therefore screening instruments for depressive symptoms, alcohol and drug consumption, and eating disorders symptoms had been included. Addressing especially pandemic-related potential additional stressors (affecting individuals via lockdown measures), the concepts of social support (20), loneliness (21), self-efficacy (22, 23), perceived stress and resilience (24) had been included in the assessment for this explorative study. Further, there is a growing literature body on medical students' mental health [e.g., (25)], but comparably little is known about other faculties' students mental health or about a comparison between faculties. Since curricular organization (e.g., requirements regarding mandatory courses and hence the impact on students' life) differ between faculties, it seems to be important to assess these parameters.

To our knowledge the present study about mental health status and COVID-19 pandemic aspects among University students in the University of Leipzig is the first in Germany–except for a survey of the University of Konstanz, Germany, which was, however, conducted with the main focus on students' experience with digital teaching (26). While in this study, the majority of the participants (74%) reported that all their planned lectures took place, the students reported both positive and negative side effects of digital teaching, with flexibility being a positive (60%) and lack of social dialogue with other students (80%) being a negative effect. The University of Leipzig with ~30,000 students and 14 faculties, ranging from medicine to theology, can be considered representative for a full-scale University in Germany and Europe. This study aims to investigate the mental health status and perceived burdens of University students during COVID-19 pandemic lockdown in March and April 2020 in Germany in an anonymous online survey. Further, potential predictors of mental health status will be investigated exploratively.

Lockdown measures in Germany in spring 2020 were comparable to other European countries, e.g., Italy, Spain, or Great Britain. Students had been asked to answer retrospectively how they experienced this time period. At the time point of the survey there were still several lockdown measures in place, such as forbidden or limited mass or group events, wearing masks, social distancing, and tests for contact person.

## Materials and Methods

### Participants and Procedure

The cross-sectional study was conducted online in July and August 2020, during the last weeks of the ongoing summer semester (and before the exam period). A total of *N* = 3,382 University students (15% of all students at the University of Leipzig) completed the online survey. The survey was set up in the online-survey-tool LimeSurvey® (Version 3.22.27) in German and English language. All students at the University were invited via email and the social media channels of the University to participate. All participants provided informed consent prior to participation. The only inclusion criterion was being currently enrolled as a student, there were no exclusion criteria. The Ethics Committee of the Medical Faculty, University of Leipzig, waived ethical approval for this study because of anonymity of the survey (06-22-2020).

### Measures

Respondents were asked about socioeconomic information (their faculty, income, and change in income, residential situation, relationship status, migration status, and being parent), chronic physical illness status, times of personal and indirect social contact and their media, and social media use. In addition, the following measures were used:

#### COVID-19 Pandemic: Attitudes and Perceived Burdens

University students were asked about their attitudes toward the COVID-19 pandemic, psychosocial consequences and estimation of individual proximity of the pandemic as well as perceived burdens due to the regulations and lockdown measures. Additionally, positive and negative aspects of the pandemic were assessed in free text format and whether or not stockpile behavior was present. Items were based on an existing questionnaire battery previously used in a cohort study and adapted for the pandemic and lockdown situation (27).

#### Mental Health Measures

Depressive symptoms over the past 2 weeks were assessed with the **Patient Health Questionnaire-9 [PHQ-9;** (28)]. Symptoms were rated on a 4-point Likert scale from 0 = “not at all” to 3 = “nearly every day.” The total sum score ranges from 0 to 27, with higher scores indicating higher levels of depressive symptoms.

Further, respondents were asked about lifetime mental disorders (psychiatric diagnoses) as well as past and current treatments for mental disorders.

Alcohol and drug consumption was assessed using the **Alcohol Use Disorders Identification Test [AUDIT-C;** (29)]. Respondents were asked to report the frequency for having alcoholic drinks on a 5-point Likert scale ranging from “never” to “4 or more times a week.” Further, they were asked about the number of standard drinks on a typical day when drinking. The subscale “Hazardous alcohol use” had been calculated. With respect to the special situation of the COVID-19 pandemic, a new item was added, asking respondents for a potential change in drinking behavior during the COVID-19 pandemic (no change, drinking less, drinking more). To assess drug consumption, the AUDIT items and the additional item on change in consumption were rephrased to “drug or substance use.”

The **Short Evaluation of Eating Disorder [SEED;** (30)] was used to investigate key eating disorder symptoms, except for weight and height. There are five items with different answer cues. “Are you afraid of becoming fat or gaining weight?” is answered on a 5-point Likert scale from 0 = “not at all” to 4 = “constantly.” Four items are asking about body perception and body image, e.g., “In what way do you perceive your body?” with a 5-point Likert scale with answers ranging from 1 = “much too thin” to 5 = “much too fat.” In the present analysis the calculation of the severity index for anorexia symptoms has not been applied, as weight and height had not been assessed. Items will be analyzed descriptively. The severity index for bulimia nervosa symptoms (BN-TSI) had been calculated.

Additionally, respondents were asked if their body weight had changed during the pandemic and if they attributed changes in body weight to the pandemic and lockdown situation.

#### Social and Emotional Aspects of the COVID-19 Pandemic

Social support was assessed using the **ENRICHED Social Support Inventory [ESSI;** (31)] with five items rated on a 5-point Likert scale from 1 = “none of the time” to 5 = “all of the time” with the total score ranging from 5 to 25. Higher scores indicate a higher level of social support.

Experienced loneliness was assessed with the **UCLA 3-Item Loneliness Scale** (32). Items are answered on a 4-point Likert scale ranging from “never” to “often,” with higher values indicating more experienced loneliness.

The **General Self-Efficacy Scale [GSE; (****33****)]** assesses with ten items the general sense of perceived self-efficacy in regards to coping with daily hassles and adaptation after experiencing stressful life events. Answering format was on a 4-point Likert scale from 1 = “not true at all” to 4 = “exactly true” to statements like: “If someone opposes me, I can find the means and ways to get what I want.” Items are added to a score from 10 to 40. Higher values indicate higher self-efficacy.

The **Brief Resilience Scale [BRS; (****34****)]** was administered to measure resistance to illness, adaptation, and thriving, the ability to bounce back or recover from stress on six items with a 5-point Likert scale from1 = “strongly disagree” to 5 = “strongly agree.” A mean score is calculated, with higher values indicating higher levels of resilience.

The **Perceived Stress Scale [PSS-4; (****35****)]** assesses with four items the extent to which individuals appraise situations in their lives as excessively uncontrollable and overloaded. Statements like “In the last month how often have you felt you were unable to control the important things in your life?” were answered on a 5-point Likert scale from 0 = “never” to 4 = “very often.” Higher sum scores indicate more perceived stress.

**Frequency of personal or indirect contacts** during the period of contact ban (March and April 2020) was measured with items developed by the research team asking to rate the frequency of contacts, e.g., “How many times per week did you personally meet with people (family members, friends, neighbors, etc.) beside your own household?” or “How many times per week did you have indirect contact, e.g., via phone, with other persons (family members, friends, neighbors, etc.) beside your own household?” on a 6-point Likert scale from 1 = “not at all” to 6 = “multiple times a day.”

Further, students were asked to report the extent to which various aspects of their lifestyle (social and cultural activity, healthy eating, dating behavior, and sexual activity) were affected by the governmental restrictions during lockdown.

### Statistical Analysis

Statistical analyses were performed using IBM SPSS Statistics version 26.0. A two-tailed α = 0.05 was applied to statistical testing. First, descriptive statistics were performed for socioeconomic variables, attitudes toward the COVID-19 pandemic and perceived burdens, mental health status measures, and ratings on social and emotional aspects of the pandemic. Second, a one-way ANOVA was performed to assess differences in depressive symptoms (PHQ-9 sum score) between three groups of students with different self-rated income changes due to the COVID-19 lockdown (no change, decreased income, and increased income).

Also, a one-way ANOVA was performed to test potential differences between students with different faculty affiliations in PHQ-9 sum score in an explorative way, as curricular organization varies substantially between faculties. An asymptotic Kruskal-Wallis-*H*-test was conducted to test if the number of students answering the PHQ-9 item 9 (suicidality) ≥1 differed between the Humanities, Medicine, Natural Science, and Social Studies faculties. Also, a *t*-test was performed to test potential differences in the mean PHQ-9 sum score between students being enrolled in two or more than two faculties and students being enrolled in only one faculty; and a Mann-Whitney-*U*-test to test potential differences in the number of students answering PHQ-9 item 9 ≥ 1 between between the aforementioned two groups.

A multiple linear regression was calculated to predict depressive symptoms (PHQ-9 sum score) based on socioeconomic variables (marital status, residential relationship, being parent), self-rated number of direct and indirect contacts, perceived stress (PSS-4), loneliness (UCLA-3), social support (ESSI), and self-efficacy (GSE). Categorical variables with more than two categories were either dichotomized (marital status, residential status, being parent) or recoded into dummy variables (direct and indirect contacts). All predictor variables were entered simultaneously. The assumption of multicollinearity was not violated (Variation Inflation Factor [VIF] ≤ 10; correlation matrix check [*r* ≤.85]). All effect sizes were interpreted as suggested by Cohen (36). Further, two multiple linear regression analyses were calculated to predict alcohol consumption and bulimia nervosa symptom severity based on socioeconomic variables (marital status, residential relationship, being parent), self-rated number of direct and indirect contacts, perceived stress (PSS-4), loneliness (UCLA-3), social support (ESSI), self-efficacy (GSE) and depressive symptoms (PHQ-9 sum score).

## Results

Sociodemographic characteristics are displayed in Table 1. Of the *N* = 3,382 participating students, 70.2% were female, 28.6% male, and 1.2% diverse with an age range of 17–61 years (*M* = 23.98, *SD* = 4.66). Next to the *N* = 3,382 participating students, *N* = 491 were drop-outs. Socioeconomic data was available of *N* = 256 of the drop-outs. There were significant differences between the drop-outs with socioeconomic data (*N* = 256) and completers (*N* = 3382) regarding migration status [16% in drop-outs, 5% in analyzed sample, χ^2^(1) = 44.117, *p* < 0.001), relationship status [59% single in drop outs vs. 50% single in analyzed sample; 33% living in a partnership in drop-outs vs. 44% living in a relationship in analyzed sample; χ^2^(5) = 64.869, *p* < 0.001]. Other socioeconomic data (age, gender, being parent, faculty enrolment, and income) did not show any significant differences.

**Table 1:** Characteristics of the total sample (*N* = 3,382).

	**(*n*, %)**	**Depressive symptoms**	Perceived stress^*^	**Social support**	**Loneliness**	**Self-efficacy**
**Total** [*M* (*SD*)]	3382	8.66 (5.46)	7.35 (3.17)	21.4 (3.7)	5.02 (2.2)	28.57 (4.46)
**Gender** [*M* (*SD*)]						
Female	2374 (70.2)	8.19 (5.47)	7.02 (3.22)	20.46 (4.15)	4.85 (2.3)	29.15 (4.54)
Male	967 (28.6)	8.78 (5.39)	7.45 (3.12)	21.8 (3.42)	5.09 (2.15)	28.38 (4.39)
Divers	41 (1.2)	12.98 (6.72)	9.27 (3.56)	20.37 (3.79)	5.54 (2.43)	26.39 (4.78)
**Age** (*M* (*SD*); years)	23.98 (4.66)					
<20	253 (7.5)	9 (5.86)	7.23 (3.3)	21.31 (3.73)	5.43 (2.29)	28.35 (4.24)
20–25	2260 (66.8)	8.7 (5.33)	7.34 (3.11)	21.59 (3.53)	5.02 (2.13)	28.55 (4.36)
26–30	612 (18.1)	8.64 (5.57)	7.48 (3.21)	21.21 (3.71)	4.93 (2.27)	28.59 (4.53)
≥31	257 (7.6)	8.04 (5.88)	7.22 (3.4)	20.28 (4.73)	4.91 (2.46)	28.98 (5.25)
**Marital status** [*M* (*SD*)]						
In a relationship	1693 (50.1)	8.11 (5.27)	7.03 (3.1)	22.66 (2.74)	4.68 (2.18)	28.9 (4.35)
Single	1689 (49.6)	9.22 (5.59)	7.66 (3.21)	20.14 (4.08)	5.36 (2.16)	28.25 (4.54)
**Residential status** [*M* (*SD*)]						
Alone	678 (20)	9.1 (5.76)	7.56 (3.32)	20.34 (4.29)	5.25 (2.26)	28.42 (4.71)
Not alone	2704 (80)	8.55 (5.38)	7.29 (3.13)	21.67 (3.48)	4.97 (2.18)	28.61 (4.39)
With children	167 (4.9)	7.51 (5.67)	6.89 (3.33)	21.88 (3.4)	4.6 (2.34)	29.68 (4.5)
Without children	3215 (95.1)	8.72 (5.44)	7.37 (3.16)	21.38 (3.71)	5.05 (2.19)	28.52 (4.45)
**Being parent** [*M* (*SD*)]						
Yes	189 (5.6)	7.26 (5.8)	6.88 (3.28)	21.72 (3.78)	4.56 (2.33)	29.94 (4.63)
No	3193 (94.4)	8.74 (5.42)	7.38 (3.16)	21.38 (3.69)	5.05 (2.19)	28.5 (4.43)
**Migration status** [*M* (*SD*)]						
Self	188 (5.6)	10.92 (6.09)	8.11 (2.98)	19.63 (4.7)	5.46 (2.17)	28.79 (4.85)
Parents	226 (6.7)	9.85 (5.56)	8.01 (3.3)	21.15 (3.64)	5.39 (2.16)	28.42 (4.56)
**Income** [*M* (*SD*)]						
***Before*** **COVID-19 lockdown**						
No income^**^	544 (16.1)	8.87 (5.64)	7.6 (3.31)	21.14 (3.94)	5.26 (2.24)	27.96 (4.44)
1–499 €/mo	658 (19.5)	8.88 (5.61)	7.53 (3.09)	21.55 (3.62)	5.19 (2.18)	28.25 (4.51)
500–1000 €/mo	1510 (44.6)	8.6 (5.23)	7.34 (3.06)	21.46 (3.53)	5 (2.13)	28.62 (4.32)
>1000 €/mo	579 (17.1)	8.31 (5.65)	7.01 (3.33)	21.45 (3.88)	4.71 (2.3)	29.44 (4.63)
No answer	91 (2.7)	9.01 (5.69)	6.96 (3.3)	20.57 (4.14)	4.82 (2.13)	28.37 (4.62)
***After*** **COVID-19lockdown**						
No income^**^	658 (19.5)	9.03 (5.61)	7.73 (3.25)	21.2 (3.88)	5.27 (2.23)	28.04 (4.54)
1–499 €/mo	736 (21.8)	8.87 (5.6)	7.58 (3.11)	21.52 (3.62)	5.07 (2.17)	28.15 (4.41)
500–1000 €/mo	1392 (41.2)	8.71 (5.27)	7.35 (3.08)	21.47 (3.54)	5.03 (2.14)	28.61 (4.31)
>1000 €/mo	505 (14.9)	7.69 (5.42)	6.59 (3.22)	21.49 (3.83)	4.63 (2.31)	29.83 (4.56)
No Answer	91 (2.7)	8.89 (5.77)	6.8 (3.25)	20.37 (4.31)	4.91 (2.13)	28.44 (4.68)
**Change in income**						
Decrease	551 (16.3)	10.23 (5.88)	8.37 (3.09)	20.93 (4.15)	5.31 (2.19)	28.15 (4.65)
No Change	2699 (79.8)	8.38 (5.32)	7.18 (3.14)	21.49 (3.61)	4.96 (2.19)	28.63 (4.41)
Increase	132 (3.9)	7.92 (5.34)	6.58 (3.15)	21.66 (3.19)	5.08 (2.25)	29.21 (4.51)

* *Reduced sample size of n = 3,379 due to missing data*.

** *Respondents indicate not to receive any own income on a regular basis, reason unknown*.

*PHQ, Patient Health Questionnaire-9 sum score; Perceived Stress, Perceived Stress Scale-4; Social Support, ENRICHED Social Support Inventory; Loneliness, University of California Los Angeles Loneliness Scale; Self-Efficacy, General Self-Efficacy Scale*.

In total, *n* = 551 (16.3%) indicated that they suffered from a chronic medical condition. Regarding their financial situation, *n* = 2,699 (79.8%) of the students reported that their financial situation during the COVID-19 pandemic remained stable, *n* = 551 (16.3%) of the respondents indicated that it had worsened, and *n* = 132 (3.9%) reported an increase. The most frequently reported reason for a decrease in income was job loss, including failure of contract renewal/extension (*n* = 380, 68.9%). The most frequently reported reason for an increase were additional or new jobs (e.g., in pandemic management) or salary increases (*n* = 105, 79.5%).

A one-way ANOVA was conducted to compare the three groups of students with different self-rated income changes (no change, decreased income, increased income) on their levels of depressive symptoms (PHQ-9 sum score). These groups showed the following PHQ-9 scores: no change: *M* = 8.38, *SD* = 5.32; decreased income: *M* = 10.23, *SD* = 5.87; and increased income: *M* = 7.92, *SD* = 5.34. The level of depressive symptoms differed significantly between the three groups, Welch's *F*_(2, 24.757)_ = 317.441, *p* < 0.001, η^2^ = 0.04. *Post-hoc*-analysis revealed that the significant main effect was based on higher PHQ-9 sum scores in the group of students reporting a decreased income. There was no significant difference between the group reporting no change and the group reporting increased income.

### COVID-19 Pandemic: Attitudes and Perceived Burdens

In total, *n* = 1,658 (49.0%) reported that they were worried or very much worried about COVID-19, whereas, almost the same number (*n* = 2,052, 60.7%) reported not to feel personally in danger at present. The vast majority of respondents (*n* = 2,859, 84.5%) supported the governmental lockdown measures.

In total, *n* = 1,594 (47.1%) agreed or fully agreed to the statement that they were being hit hard by the COVID-19 pandemic and that governmental lockdown measures hit students particularly hard (*n* = 1,472, 43.5%). Almost all respondents (*n* = 3,243, 95.9%) disagreed with the statement that the pandemic was part of a larger conspiracy.

*N* = 427 (12.6%) reported stockpiling behavior when grocery shopping. *N* = 1,679 (49.6%) indicated to know at least one person being infected with COVID-19.

During the lockdown period in Germany, *n* = 1,572 (46.5%) did report not to have any direct personal contact to other people (beyond the ones living together), *n* = 1,339 (39.6%) indicated to have had personal contact one or two times per week and *n* = 471 (13.9%) three times or more per week; *n* = 2,813 (83.2%) reported to have indirect contact (e.g., phone or video calls) seven times or more per week.

Overall, *n* = 2,541 (75.1%) of the students indicated that they had experienced positive aspects during the COVID-19 pandemic, *n* = 3,150 (93.1%) reported that they experienced negative aspects. As positive aspects of COVID-19 pandemic on their personal life, more free time, less social pressure, higher flexibility due to digital teaching, less air pollution (as positive side effect of lockdown measures), more time to focus and reflect on “what really matters in life,” were mentioned the most. The following negative aspects of COVID-19 pandemic on personal life were mentioned the most: financial problems/unemployment, missing friends and family, uncertainty of the future, impossibility to travel for vacation purposes/missing national and international traveling in general, and impossibility to travel for studying purposes (international internship or semester abroad).

### Mental Health Measures

More than one third of the students reported a PHQ-9 sum score of 10 or above, indicating clinically relevant symptoms (*n* = 1,249, 37.0%). The PHQ-9 sum score of the total sample was on average 8.66 (*SD* = 5.46). Almost every seventh student reported suicidal thoughts on at least several days per week over the past 2 weeks (*n* = 490, 14.5%).

*N* = 644 (19.0%) of the students reported to have been diagnosed with a mental disorder in the past: unipolar depression (*n* = 334, 9.9%), bipolar disorder (*n* = 12, 0.4%), anxiety disorder (*n* = 228, 6.7%), obsessive-compulsive disorder (*n* = 46, 1.4%), personality disorder (*n* = 62, 1.8%), eating disorder (*n* = 127, 3.8%), and others (*n* = 93, 2.7%).

Of all students reporting a mental disorder (*n* = 644), half of them (*n* = 378, 58.7%) indicated not to receive any treatment, whereas *n* = 56 (8.7%) currently took medication, *n* = 145 (22.5%) were in psychotherapeutic treatment, and *n* = 65 (10.1%) reported to take both medication and receiving psychotherapy.

A substantial amount of the students (*n* = 1,130, 33.0%) reported binge eating at least once per week, *n* = 117 (3.5%) reported vomiting as compensatory behavior minimum once per week, *n* = 34 (0.1%) usage of laxative minimum once per week, *n* = 844 diet or calorie food (25.0%) minimum once per week, and *n* = 941 (28.0%) reported excessive exercising minimum once per week. The bulimia nervosa severity index (BN-TSI, SEED) of the sample was *M* = 0.35 (*SD* = 0.44) (*N* = 3,380). Further, *n* = 1,548 (45.8%) reported weight changes during the COVID-19 lockdown, *n* = 885 (26.2%) reported weight gain, *n* = 663 (19.6%) weight loss. Of those reporting any weight change, over 63% attributed this to the pandemic and lockdown.

Reported alcohol and drug consumption are displayed in Table 2.

**Table 2:** Alcohol and drug consumption and changes during COVID-19 pandemic.

**Variable**	***N* = 3382**
**Current alcohol consumption** (*n*, %)	
Abstinent	612 (18.1)
≤1 per month	707 (20.9)
2–4 times a month	1133 (33.5)
2–3 times a week	788 (23.3)
4 or more times a week	142 (4.2)
**Change in drinking behavior during the COVID-19 pandemic** (*n*, %)	
Less	821 (29.6)
Equal	1314 (47.5)
More	634 (22.9)
**AUDIT-C subscale hazardous alcohol use** [MW (SD)], *N* = 2,769	3.5 (1.86)
**Current drug or substance use** (*n*, %)	
Abstinent	2818 (83.3)
≤1 per month	303 (9.0)
2–4 times a month	144 (4.3)
2–3 times a week	50 (1.5)
4 or more times a week	66 (2.0)
**Change in drug/substance use during the COVID-19 pandemic** (*n*, %)	
Less	153 (27.2)
Equal	246 (43.7)
More	164 (29.1)

To investigate potential differences in faculty affiliation and PHQ-9 sum score, a one-way ANOVA was conducted to compare students of the four major faculty groups (Humanities, Medicine, Natural Science, and Social Studies) regarding their levels of depressive symptoms (PHQ-9 sum score). The following mean PHQ-9 sum scores were found: Humanities *M* = 10.06 (*SD* = 5.65), Medicine *M* = 6.64 (*SD* = 4.94), Natural Science *M* = 8.84 (*SD* = 5.57), and Social Studies *M* = 8.4 (*SD* = 5.23). The level of depressive symptoms differed significantly between the four groups, Welch's *F*_(4, 1193.105)_ = 42.995, *p* < 0.001, η^2^ = 0.09. Further, an asymptotic Kruskal-Wallis-*H*-test was conducted to test if the number of students answering the PHQ-9 item 9 on suicidal thoughts ≥1 (see Table 3) differed between the Humanities, Medicine, Natural Science, and Social Studies faculties. There was a statistically significant difference in the number of students answering PHQ-9 item 9 ≥ 1 between the four groups, *H* = 29.018, *p* < 0.001 (see Table 3).

**Table 3:** University faculties, PHQ-9 sum score and PHQ-9 item 9 on suicidal thoughts.

**Faculty (*n*, %)**	**PHQ-9 sum score [*M* (*SD*)]**	PHQ Item 9^*^ ≥ 1 (*n*, *%*)
**Total (4076, 100)**	8.66 (5.46)	566 (100)
**Humanities (1309, 26.6)**	10.06 (5.65)	217 (38.3)
History, Arts and Oriental Studies (349, 8.6)	9.63 (5.75)	66 (30.4)
Law (195, 4.8)	9.13 (5.57)	27 (12.4)
Philology (676, 16.6)	9.76 (5.52)	115 (53)
Theology (89, 2.2)	8.88 (5.38)	9 (4.1)
**Medicine (428, 12.8)**	6.64 (4.94)	40 (7.1)
Medicine (341, 8.4)	6.6 (4.83)	29 (72.5)
Veterinary medicine (87, 2.1)	6.91 (5.39)	11 (27.5)
**Natural Science (619, 13.9)**	8.84 (5.57)	86 (15.2)
Chemistry und Mineralogy (119, 2.9)	8.18 (5.02)	14 (16.3)
Mathematics and Computer Science (280, 6.9)	8.44 (5.45)	40 (46.5)
Physics und Earth Sciences (220, 5.4)	8.68 (5.94)	32 (37.2)
**Social Studies (1720, 46.72)**	8.4 (5.23)	223 (40.1)
Economics and Management Science (207, 5.1)	8.05 (5.22)	24 (10.8)
Education (749, 18.4)	8.34 (5.29)	79 (35.4)
Life science (241, 5.9)	8.06 (5,31)	40 (17.9)
Social Science und Philosophy (396, 9.7)	9.2 (5.43)	71 (31.8)
Sports Science (127, 3.1)	6.75 (4.3)	9 (4)
**Enrollment≥ 2 faculties (505, 12.4)**	8.15 (5.38)	55 (10.9)

* “Thoughts that you would be better off dead, or of hurting yourself.” 0 = “Not at all,” 1 = “Several days,” 2 = “More than half the days,” and 3 = “Nearly every day.”

An exploratory multiple regression analysis examined predictors of depressive symptomatology (see Table 4). Not being a parent (*p* = 0.010), having no indirect contact one or two times a week (*p* = 0.005), higher perceived stress (*p* < 0.001), higher experienced loneliness (*p* < 0.001), lower social support (*p* < 0.001), and lower self-efficacy (*p* < 0.001) significantly predicted higher levels of depressive symptoms. All other predictors were unrelated to depressive symptoms (all *p* > 0.05). The overall model fit was *R*^2^ = 0.523 (adjusted *R*^2^ = 0.521).

**Table 4:** Linear regression analysis for predictors of depressive symptoms (PHQ-9).

	**Total Sample (*****n*** **= 3,379)**					
**Variable**	**Unstandardized B**	**SE B**	**Standardized *B***	**95% Confidence Interval (CI)**	***t***	***p***
Marital status^a^	−0.277	0.143	−0.025	−0.004,0.557	−1.933	0.053
Residential status^b^	−0.044	0.167	−0.003	−0.371,0.283	−0.265	0.791
Being parent	−0.754	0.291	−0.032	−0.183, −1.325	−2.591	**0.010**
Direct contact^d^						
0 times a week	0.048	0.200	0.004	0.440, −0.343	−0.242	0.809
1–2 times a week	−0.267	0.203	−0.024	−0.665,0.189	−1.315	0.189
Indirect contact^e^						
0 times a week	0.175	0.589	0.004	−0.980, 1.329	0.296	0.767
1–2 times a week	−0.507	0.181	−0.034	−0.861. −0.153	−2.805	**0.005**
Perceived stress	0.826	0.026	0.026	0.776,0.877	31.849	**<0.001**
Loneliness	0.569	0.036	0.036	0.497,0.640	15.600	**<0.001**
Social support	−0.141	0.021	−0.096	−0.183, −0.100	−6.713	**<0.001**
Self-efficacy	−0.118	0.017	−0.096	−0.152, −0.084	−6.854	**<0.001**
*R*^2^ (*R*^2^ adjusted)	0.523 (0.521)					
*F*	335.363					
*P*	**<0.001**					

a Marital status dichotomised into “single” and “in a relationship.”

b Residential status dichotomised into “alone” and “not alone.”

c Being parent dichotomised into “being parent” and “not being parent.”

d *Direct contact with the categories 0, 1–2 and ≥3 times a week*.

e *Indirect contact with the categories 0, 1–2 and ≥3 times a week*.

*Bold font indicates statistical significance, p < .05*.

Further, two multiple regression analyses examined predictors of reported alcohol consumption (AUDIT-C, hazardous alcohol use subscale) and predictors of bulimia nervosa severity index (BN-TSI, SEED) (see Supplementary Tables A,B, Supplementary Material). Not being a parent (*p* < 0.001), having no direct contact (*p* < 0.001) or direct contact one or two times a week (*p* = 0.01), no indirect contact (*p* = 0.16), higher perceived stress (*p* = 0.001), higher experienced loneliness (*p* < 0.001), lower social support (*p* < 0.001), lower self-efficacy (*p* = 0.001) and higher depressive symptoms (*p* = 0.004) significantly predicted higher levels of reported alcohol consumption. All other predictors were unrelated to reported alcohol consumption (all *p* > 0.05). The overall model fit was R^2^ = 0.061 (adjusted R^2^ = 0.06). Not being a parent (*p* = 0.018), higher perceived stress (*p* = 0.001), lower self-efficacy (*p* = 0.007) and higher depressive symptoms (*p* < 0.001) significantly predicted higher severity of bulimia nervosa related symptoms. All other predictors were unrelated to bulimia nervosa severity index (all *p* > 0.05). The overall model fit was R^2^ = 0.206 (adjusted R^2^ = 0.203).

### Social and Emotional Aspects of the COVID-19 Pandemic

Descriptive statistics of perceived stress, social support, loneliness, and self-efficacy are displayed in Table 1.

When being asked how much the restrictions affected certain aspects of lifestyle, the majority of students rated that their social (*n* = 2,357, 80.2%) and cultural activities (*n* = 2,662, 90.7%), hobbies (*n* = 1,302, 44.3), and dating behavior or partner search (*n* = 2,208, 75.2%) were significantly restricted during the COVID-19 pandemic as compared to before. Restrictions were rated not to affect or not being relevant for the following areas: healthy eating (*n* = 2,693, 91.7%) and sexual activity (*n* = 1,716, 58.3%).

Regarding media use, the internet and social networks were rated to be used daily by *n* = 2,346 (69.4%) of the students, music streaming services by *n* = 1,206 (35.7%), and video streaming by *n* = 356 (10.5%) on a daily basis. Reported usage of messenger services (daily by *n* = 3,088, 91.3% of the respondents) was evaluated as increased during the pandemic.

## Discussion

The 3,382 participants in this online survey provide a unique insight into mental health, attitudes and social and emotional aspects regarding COVID-19 pandemic of University students.

It can be summarized based on the results, that the University students in this sample seem to cope adaptively with the pandemic and drastically changed life circumstances. Nevertheless, the COVID-19 pandemic appears to be a significant burden and students seem to suffer from lockdown measures.

The sample can be considered as representative for German University students, as the University of Leipzig (second oldest University in Germany) is a large full-scale University, providing an array of all faculties and a remarkable sample size. Existing differences between drop-outs and completers can be considered as not relevant for the performed analysis and not hampering the generalizability of the results. Minor differences between the analyzed sample and all University students exist regarding gender (70% female in this sample vs. 60% female students being enrolled at the University of Leipzig) and migration status [5% vs. 11% of the students enrolled (37) statista.com, 2018]. The University of Leipzig has, compared to the average of other Universities in Germany more female students (38). Further, the students in the present sample reported more income, especially in the category 500–1000 Euro compared to German University students overall (34% in Germany vs. 41% in the present sample) (39). Further, the mental health status of this sample seems highly comparable to previous epidemiological studies in this population, which estimate that 12–46 % of all University students are affected by mental disorders in any given year (40, 41).

For the majority of the students (80%) the financial situation did not change due to the pandemic. Nevertheless, for those where it deteriorated, the consequences might be extensive. *Post-hoc* analysis revealed a significant difference in depressive symptoms in this sample for the three groups of students: where the income had stayed stable, income had increased, and income had decreased. Next to a major change in social life due to governmental restrictions, financial challenges seem to be a major stress factor for students indeed. It has long been known that part-time jobs (next to state funding and parental support) play a key role in financing the livelihood of students (42). But, during the lock-down situation in Germany over 800.000 part-time jobs were abolished. About 120,000 students applied for state funded emergency aid between June and September of 2020 (43). The pandemic certainly took a major toll on the financial situation of students, next to other issues. Consistent with present results a job loss can lead to lower psychological well-being (44).

### COVID-19 Pandemic: Attitudes and Perceived Burdens

Half of the students in this survey reveal that they are worried or very much worried about the COVID-19 pandemic, but not feeling in personal danger. This mirrors the overall picture in most European countries during spring and summer 2020.

Also, half of the sample frankly indicated that the perceived burden by the pandemic and the lockdown measures was very high. It seems that direct and indirect contacts had been restricted tremendously, as governmental restrictions have prescribed.

### Mental Health Measures

The level of depressive symptoms in this sample is comparable to recent instigations of University students in other countries (45), or even higher in this sample compared to other studies (46), whereas, the sample size here was enormously smaller (*N* = 369) (46).

The mean PHQ-9 sum score in this sample is to be considered as status with mild depressive symptoms (PHQ-9 sum score between 5 and 9), above 10 is considered to represent clinically relevant depressive symptoms (28). Every seventh student in this sample reported suicidal thoughts over the past 2 weeks on at least several days, this is comparable to other studies in this population [e.g., (47)], but remarkably higher than in the general population (48). Students in the Humanities faculties scored highest on depressive symptoms and on suicidal ideation as well, medical students the lowest.

Further, half of the students who indicated that they had been diagnosed with any mental disorder in the past, reveal that they did not receive any treatment. This probably also holds true for all subclinical forms of mental disorders (e.g., in this sample sublinical levels of depressive symptoms and a substantial, (but comparable to other studies) amount of subclinical eating disorder symptoms (49). This substantial gap between present symptoms of mental disorders and received treatment could be filled by available low-threshold online interventions, especially during this pandemic (46).

Regarding alcohol and drug consumption, the results reveal that a substantial proportion (appr. 80 %) of the respondents indicate that they consumed less or equal amounts, which could be explained by fewer occasions (e.g., parties or events) and potential places to meet (e.g., bars and restaurants) due to lockdown measures and safety issues. Next to this, every 5th student of the sample reported that they drank more alcohol, presumably alone and serving as a compensatory means during a time of contact bans and reduced social activities. This could eventually lead to alcohol dependency in the medium or long-term for a subset of individuals and should therefore be addressed by preventive (online) efforts.

The regression analyses surprisingly revealed that being a parent seems to be a protective factor for depressive symptom load, alcohol consumption and some eating disorder symptoms in University students. This contradicts some widespread beliefs that it is a dual burden for parents in general to work/study in home office and to take care of children, when day care and schools are being closed. Marital status (being in a relationship vs. not) and residential status (living alone vs. living in a shared flat) seem not to be associated with depressive symptoms, alcohol consumption and bulimia nervosa symptoms. As expected, perceived high stress, high experienced loneliness, low rated social support and fewer self-efficacy beliefs are predicting higher depressive symptoms, also higher alcohol consumption and higher eating disorder symptoms in this sample. Also, neither form of contact (direct and indirect) beyond the people living in the same household and close family seem to be associated with higher depressive symptoms, which is in line with the main-effect and buffering model in the social support theory by Cohen (20, 50). The main effect model assumes a generally positive influence of social integration on psychological well-being (20). In contrast, the buffering effect model assumes a mitigating effect of the consequences of stress, through social support, in times of high psychological stress.

Even without a global pandemic, students' mental health care situation seems challenging (16, 51–53) with several unmet needs (54), it is even described as a “student mental health crisis” in some countries (55). Nevertheless, this pandemic could be an opportunity to improve mental health services as well (56).

### Social and Emotional Aspects of the COVID-19 Pandemic

Levels of perceived stress, social support, loneliness, and self-efficacy were all elevated, but comparable to other studies in University students [e.g., (46) reporting elevated PSS-4 scores >8 in half of the sample of US students]. University students are being hit hard in their daily social life by lockdown measures, as they prevent the majority of social experiences. They are in turn essential for perceived autonomy, competence, and relatedness. A lack of these important components of self-determination might lead to decreased well-being (57), under non-pandemic conditions and is present in this study presumably under pandemic-conditions as well.

Further, under non-pandemic conditions peer support, social support (58, 59) and self-efficacy (60) are next to other factors crucial for general well-being, life satisfaction, and also academic performance (60–62) especially in University students. Due to the lockdown measures and social distancing, especially the peer and social support (rather than family support) dropped out, which can explain the present results. As expected, the respondents in this sample reported a medium to high usage of social media and online resources, also an increased usage of IT technologies (as also expected and prominent in the majority of the general public) during the pandemic. Next to the advantages of an increased “being online,” the lockdown with the attached loss of social contacts and a regular daily routine, the digitalization of University teaching has been and still represents an unprecedented challenge for students.

## Limitations

The present study has a remarkably high sample size and applies to a vast majority standardized measurements. Due to the cross-sectional study design, no causal inferences can be drawn. Several statistical tests had been applied, but due to the explorative character of the analysis multiple testing is considered not to be an issue.

## Conclusion

University students are, besides a general adaptive coping with the COVID-19 pandemic and the associated lockdown, a vulnerable group and are at risk for mental disorders. This study implies that Universities and health care providers need to take action to continuously assess, prevent, identify, and manage mental health conditions of University students in an adequate manner, as other studies among University students in China claimed already (45). Supporting the health, mental health and well-being of all students should be of high priority in pandemic and post-pandemic times (63). Online programs, especially focusing on help-seeking behavior (64), chat interventions (e.g., with messenger services) and other low-threshold support programs or self-management interventions seem promising, easy to implement and are evidence-based for nearly all common mental health conditions.

## Data Availability Statement

The raw data supporting the conclusions of this article will be made available by the authors upon reasonable request, without undue reservation.

## Ethics Statement

The Ethics Committee of the Medical Faculty, University of Leipzig, waived ethical approval for this study because of anonymity of the survey (22.06.2020). The patients/participants provided their written informed consent to participate in this study.

## Author Contributions

EK, SB, and CR-K designed the study. RM, SB, and EK performed the statistical analysis. S-LK organized the recruitment and contributed to the literature overview. EK, RM, SB, S-LK, and CR-K discussed the results and contributed to the final manuscript. All authors have approved the final manuscript.

## Conflict of Interest

CR-K received lecture honoraria from Recordati and Servier outside and independent of the submitted work. The remaining authors declare that the research was conducted in the absence of any commercial or financial relationships that could be construed as a potential conflict of interest.

## References

[1] Kessler RC Berglund P Demler O Jin R Merikangas KR Walters EE. Lifetime prevalence and age-of-onset distributions of DSM-IV disorders in the National Comorbidity Survey Replication. Arch Gen Psychiatry. (2005) 62:593–602. 10.1001/archpsyc.62.6.593 15939837

[2] Denovan A Macaskill A. Stress and subjective well-being among first year uk undergraduate students. J Happiness Stud. (2017) 18:505–25. 10.1007/s10902-016-9736-y

[3] Tran A Tran L Geghre N Darmon D Rampal M Brandone D. Health assessment of French University students and risk factors associated with mental health disorders. PLoS ONE. (2017) 12:e0188187. 10.1371/journal.pone.0188187 29176864 PMC5703533

[4] Williams CJ Dziurawiec S Heritage B. More pain than gain: effort–reward imbalance, burnout, and withdrawal intentions within a University student population. J Educat Psychol. (2018) 110:378–94. 10.1037/edu0000212

[5] Yikealo D Yemane B Karvinen I. The level of academic and environmental stress among college students: a case in the college of education. JSS. (2018) 06:40–57. 10.4236/jss.2018.611004

[6] Heckman S Lim H Montalto C. Factors related to financial stress among college students. J Financ Ther. (2014) 5:19–39. 10.4148/1944-9771.1063

[7] Tosevski DL Milovancevic MP Gajic SD. Personality and psychopathology of University students. Curr Opin Psychiatry. (2010) 23:48–52. 10.1097/YCO.0b013e328333d625 19890212

[8] Wang C Horby PW Hayden FG Gao GF. A novel coronavirus outbreak of global health concern. The Lancet. (2020) 395:470–3. 10.1016/S0140-6736(20)30185-9 31986257 PMC7135038

[9] World Health Organization. Coronavirus Disease (COVID-19) Outbreak. Geneva: WHO (2020).

[10] Grubic N Badovinac S Johri AM. Student mental health in the midst of the COVID-19 pandemic: a call for further research and immediate solutions. Int J Soc Psychiatry. (2020) 66:517–8. 10.1177/0020764020925108 32364039 PMC7405631

[11] Wang C Pan R Wan X Tan Y Xu L McIntyre RS. A longitudinal study on the mental health of general population during the COVID-19 epidemic in China. Brain Behav Immun. (2020) 87:40–8. 10.1016/j.bbi.2020.04.028 32298802 PMC7153528

[12] Jia R Ayling K Chalder T Massey A Broadbent E Coupland C. Mental health in the UK during the COVID-19 pandemic: cross-sectional analyses from a community cohort study. BMJ Open. (2020) 10:e040620. 10.1136/bmjopen-2020-040620 32933965 PMC7493070

[13] Cao W Fang Z Hou G Han M Xu X Dong J. The psychological impact of the COVID-19 epidemic on college students in China. Psychiatry Res. (2020) 287:112934. 10.1016/j.psychres.2020.112934 32229390 PMC7102633

[14] Akdeniz G Kavakci M Gozugok M Yalcinkaya S Kucukay A Sahutogullari B. A survey of attitudes, anxiety status, and protective behaviors of the University students during the COVID-19 outbreak in Turkey. Front Psychiatry. (2020) 11:695. 10.3389/fpsyt.2020.00695 32760303 PMC7373786

[15] Kaparounaki CK Patsali ME Mousa D-PV Papadopoulou EV Papadopoulou KK Fountoulakis KN. University students' mental health amidst the COVID-19 quarantine in Greece. Psychiatry Res. (2020) 290:113111. 10.1016/j.psychres.2020.113111 32450416 PMC7236729

[16] Rahmadiana M Karyotaki E Schulte M Ebert DD Passchier J Cuijpers P. Transdiagnostic internet intervention for indonesian University students with depression and anxiety: evaluation of feasibility and acceptability. JMIR Ment Health. (2021) 8:e20036. 10.2196/20036 33666553 PMC7980121

[17] Said D Kypri K Bowman J. Risk factors for mental disorder among University students in Australia: findings from a web-based cross-sectional survey. Soc Psychiatry Psychiatr Epidemiol. (2013) 48:935–44. 10.1007/s00127-012-0574-x 22945366

[18] Fawzy M Hamed SA. Prevalence of psychological stress, depression and anxiety among medical students in Egypt. Psychiatry Res. (2017) 255:186–94. 10.1016/j.psychres.2017.05.027 28575777

[19] Lun KW Chan CK Ip PK Ma SY Tsai WW Wong CS. Depression and anxiety among University students in Hong Kong. Hong Kong Med J. (2018) 24:466–72. 10.12809/hkmj176915 30245480

[20] Cohen S. Social relationships and health. Am Psychol. (2004) 59:676–84. 10.1037/0003-066X.59.8.676 15554821

[21] Smith BJ Lim MH. How the COVID-19 pandemic is focusing attention on loneliness and social isolation. Public Health Res Pract. (2020) 30:3022008. 10.17061/phrp3022008 32601651

[22] Richardson M Abraham C Bond R. Psychological correlates of University students' academic performance: a systematic review and meta-analysis. Psychol Bull. (2012) 138:353–87. 10.1037/a0026838 22352812

[23] Klassen RM Klassen JR. Self-efficacy beliefs of medical students: a critical review. Perspect Med Educ. (2018) 7:76–82. 10.1007/s40037-018-0411-3 29484552 PMC5889382

[24] Vinkers CH van Amelsvoort T Bisson JI Branchi I Cryan JF Domschke K. Stress resilience during the coronavirus pandemic. Eur Neuropsychopharmacol. (2020) 35:12–6. 10.1016/j.euroneuro.2020.05.003 32446705 PMC7211573

[25] Schwenk TL Davis L Wimsatt LA. Depression, stigma, and suicidal ideation in medical students. JAMA. (2010) 304:1181–90. 10.1001/jama.2010.1300 20841531

[26] Lörz M Marczuk A Zimmer L Multrus F Buchholz S. Studieren unter Corona - Bedingungen: Studierende bewerten das erste Digitalsemester. Deutsches Zentrum für Hochschul- und Wissenschaftsforschung (DZHW) (2020).

[27] Förster F Stein J Löbner M Pabst A Angermeyer MC König H-H. Loss experiences in old age and their impact on the social network and depression-results of the Leipzig Longitudinal Study of the Aged (LEILA 75+). J Affect Disord. (2018) 241:94–102. 10.1016/j.jad.2018.07.070 30107351

[28] Kroenke K Spitzer RL Williams JB. The PHQ-9: validity of a brief depression severity measure. J Gen Intern Med. (2001) 16:606–613. 10.1046/j.1525-1497.2001.016009606.x 11556941 PMC1495268

[29] Higgins-Biddle JC Babor TF. A review of the alcohol use disorders identification test (AUDIT), AUDIT-C, and USAUDIT for screening in the United States: Past issues and future directions. Am J Drug Alcohol Abuse. (2018) 44:578–86. 10.1080/00952990.2018.1456545 29723083 PMC6217805

[30] Bauer S Winn S Schmidt U Kordy H. Construction, scoring and validation of the Short Evaluation of Eating Disorders (SEED). Eur Eat Disord Rev. (2005) 13:191–200. 10.1002/erv.637

[31] Kendel F Spaderna H Sieverding M Dunkel A Lehmkuhl E Hetzer R. Eine deutsche Adaptation des ENRICHD Social Support Inventory (ESSI). Diagnostica. (2011) 57:99–106. 10.1026/0012-1924/a000030

[32] Hughes ME Waite LJ Hawkley LC Cacioppo JT. A short scale for measuring loneliness in large surveys: results from two population-based studies. Res Aging. (2004) 26:655–72. 10.1177/0164027504268574 18504506 PMC2394670

[33] Schwarzer R Jerusalem M. Skalen zur Erfassung von Lehrer- und Schülermerkmalen. Dokumentation der psychometrischen Verfahren im Rahmen der Wissenschaftlichen Begleitung des Modellversuchs Selbstwirksame Schulen.: Testbeschreibung SWE. Berlin: Freie Universität Berlin. Available online at: http://www.fu-berlin.de/gesund/ (1999). p. 4

[34] Smith BW Dalen J Wiggins K Tooley E Christopher P Bernard J. The brief resilience scale: assessing the ability to bounce back. Int J Behav Med. (2008) 15:194–200. 10.1080/10705500802222972 18696313

[35] Cohen S Kamarck T Mermelstein R. A global measure of perceived stress. J Health Soc Behav. (1983) 24:385. 10.2307/2136404 6668417

[36] Cohen J. Statistical Power Analysis for the Behavioral Sciences. New York, NY: Routledge (2013). 10.4324/9780203771587

[37] Universität Leipzig. Jahresbericht der Universität Leipzig 2019 (2020). Available online at: https://tools.uni-leipzig.de/publikationen/jahresbericht/2019/#116

[38] Statistisches Bundesamt. Frauen-anteile der Studierenden, Absol-venten und des Personals an Hoch-schulen. (2020). Available online at: https://www.destatis.de/DE/Themen/Gesellschaft-Umwelt/Bildung-Forschung-Kultur/Hochschulen/Tabellen/frauenanteile-akademischelaufbahn.html (accessed February 16, 2021)

[39] VuMA. Verbrauchs- und Medienanalyse-VuMA 2021. (2021). Available online at: https://www.vuma.de/vuma-praxis/die-studie/ (accessed February 16, 2021)

[40] Auerbach RP Mortier P Bruffaerts R Alonso J Benjet C Cuijpers P. WHO World Mental Health Surveys International College student project: prevalence and distribution of mental disorders. J Abnorm Psychol. (2018) 127:623–38. 10.1037/abn0000362 30211576 PMC6193834

[41] Harrer M Adam SH Baumeister H Cuijpers P Karyotaki E Auerbach RP. Internet interventions for mental health in University students: a systematic review and meta-analysis. Int J Methods Psychiatr Res. (2019) 28:e1759. 10.1002/mpr.1759 30585363 PMC6877279

[42] Müller U. BAföG, Stipendien & Co.: Studienfinanzierung 2020 im CHECK. Centrum für Hochschulentwicklung. Available online at: https://www.che.de/2020/bafoeg-stipendien-co-studienfinanzierung-2020-im-check/.

[43] dpa. Fast eine Milliarde Euro Schulden in Corona-Krise: Studienkredite. Forschung und Lehre (2020).

[44] Brand JE. The far-reaching impact of job loss and unemployment. Annu Rev Sociol. (2015) 41:359–75. 10.1146/annurev-soc-071913-043237 26336327 PMC4553243

[45] Liu J Zhu Q Fan W Makamure J Zheng C Wang J. Online mental health survey in a medical college in China during the COVID-19 outbreak. Front Psychiatry. (2020) 11:459. 10.3389/fpsyt.2020.00845 32574242 PMC7237734

[46] Aiyer A Surani S Gill Y Iyer R Surani Z. Mental health impact of Covid-19 on students in the USA: a cross-sectional web-based survey. J Depress Anxiety. (2020) 9:375. 10.35248/2167-1044.20.9.375

[47] Mortier P Auerbach RP Alonso J Bantjes J Benjet C Cuijpers P. Suicidal thoughts and behaviors among first-year college students: results from the WMH-ICS project. J Am Acad Child Adolesc Psychiatry. (2018) 57:263–73.e1. 10.1016/j.jaac.2018.07.723 29588052 PMC6444360

[48] Garlow SJ Rosenberg J Moore JD Haas AP Koestner B Hendin H. Depression, desperation, and suicidal ideation in college students: results from the American Foundation for Suicide Prevention College Screening Project at Emory University. Depress Anxiety. (2008) 25:482–8. 10.1002/da.20321 17559087

[49] Harrer M Adam SH Messner E-M Baumeister H Cuijpers P Bruffaerts R. Prevention of eating disorders at Universities: a systematic review and meta-analysis. Int J Eat Disord. (2020) 53:813–33. 10.1002/eat.23224 31943298

[50] Cohen S Wills TA. Stress, social support, and the buffering hypothesis. Psychol Bull. (1985) 98:310–57. 10.1037/0033-2909.98.2.310 3901065

[51] Lattie EG Lipson SK Eisenberg D. Technology and college student mental health: challenges and opportunities. Front Psychiatry. (2019) 10:246. 10.3389/fpsyt.2019.00246 31037061 PMC6476258

[52] Prince JP. University student counseling and mental health in the United States: Trends and challenges. Ment Health Prev. (2015) 3:5–10. 10.1016/j.mhp.2015.03.00131037061

[53] Baik C Larcombe W Brooker A. How Universities can enhance student mental wellbeing: the student perspective. High Educ Res Dev. (2019) 38:674–87. 10.1080/07294360.2019.1576596

[54] Bruffaerts R Mortier P Auerbach RP Alonso J La Hermosillo De Torre AE. Lifetime and 12-month treatment for mental disorders and suicidal thoughts and behaviors among first year college students. Int J Methods Psychiatr Res. (2019) 28:e1764. 10.1002/mpr.1764 30663193 PMC6877191

[55] Samira Shackle. The Way Universities Are Run is Making Us Ill': Inside the Student Mental Health Crisis: A Surge in Anxiety and Stress Is Sweeping UK Campuses. What is Troubling Students, and Is It the Universities' Job to Fix It? (2019). Available online at: https://www.theguardian.com/society/2019/sep/27/anxiety-mental-breakdowns-depression-uk-students (accessed February 16, 2021)

[56] Moreno C Wykes T Galderisi S Nordentoft M Crossley N Jones N. How mental health care should change as a consequence of the COVID-19 pandemic. The Lancet Psychiatry. (2020) 7:813–24. 10.1016/S2215-0366(20)30307-2 32682460 PMC7365642

[57] Reis HT Sheldon KM Gable SL Roscoe J Ryan RM. Daily well-being: the role of autonomy, competence, and relatedness. Pers Soc Psychol Bull. (2000) 26:419–35. 10.1177/0146167200266002

[58] Jhang F-H. Life satisfaction trajectories of junior high school students in poverty: Exploring the role of self-efficacy and social support. J Adolesc. (2019) 75:85–97. 10.1016/j.adolescence.2019.07.011 31376780

[59] Wang L Liang L Liu Z Yuan K Ju J Bian Y. The developmental process of peer support networks: the role of friendship. Front Psychol. (2021) 12:615148. 10.3389/fpsyg.2021.615148 33584478 PMC7875894

[60] Freire C Ferradás MD Regueiro B Rodríguez S Valle A Núñez JC. Coping strategies and self-efficacy in University students: a person-centered approach. Front Psychol. (2020) 11:841. 10.3389/fpsyg.2020.00841 32508707 PMC7248269

[61] Li J Han X Wang W Sun G Cheng Z. How social support influences University students' academic achievement and emotional exhaustion: The mediating role of self-esteem. Lear Individ Dif. (2018) 61:120–6. 10.1016/j.lindif.2017.11.016

[62] Marôco J Assunção H Harju-Luukkainen H Lin S-W Sit P-S Cheung K-C. Predictors of academic efficacy and dropout intention in University students: Can engagement suppress burnout? PLoS ONE. (2020) 15:e0239816. 10.1371/journal.pone.0239816 33119598 PMC7595383

[63] Dodd RH Dadaczynski K Okan O McCaffery KJ Pickles K. Psychological wellbeing and academic experience of University students in Australia during COVID-19. Int J Environ Res Public Health. (2021) 18:866. 10.3390/ijerph18030866 33498376 PMC7908219

[64] Liang S-W Chen R-N Liu L-L Li X-G Chen J-B Tang S-Y. The psychological impact of the COVID-19 epidemic on guangdong college students: the difference between seeking and not seeking psychological help. Front Psychol. (2020) 11:2231. 10.3389/fpsyg.2020.02231 33013582 PMC7499802

