# Supplementary material for: Mental Health, Social and Emotional Well-Being, and Perceived Burdens of University Students During COVID-19 Pandemic Lockdown in Germany
Source: Front Psychiatry. 2021 Apr 6;12:643957. doi: 10.3389/fpsyt.2021.643957 (PMC8055863; doi:10.3389/fpsyt.2021.643957)
Supplement: Supplementary file 1 [file Data_Sheet_1.docx]

**Table A, supplementary material.** Linear regression analysis for predictors of alcohol consumption (AUDIT-C, Hazardous Alcohol Use)

|  |  | Total Sample (*n* = 2,767) | | | | | |
| --- | --- | --- | --- | --- | --- | --- | --- |
| Variable | Unstan-dardized B | | SE B | Standar-dized  *Β* | 95% Confidence Interval (CI) | *t* | *p* |
| Marital status^a^ | .113 | | .075 | .03 | -0.35, .260 | 1.497 | .134 |
| Residential status^b^ | -.114 | | .09 | -.024 | -.289, .062 | -1.268 | .205 |
| Being parent | -.860 | | .170 | -.096 | -1.193, -.528 | -5.072 | **<.001** |
| Direct contact^d^ |  | |  |  |  |  |  |
| 0 times a week | -.715 | | .105 | -.190 | -.921, -.509 | -6.816 | **<.001** |
| 1 - 2 times a week | -.271 | | .105 | -.072 | -.477, -.065 | -2.578 | **.01** |
| Indirect contact^e^ |  | |  |  |  |  |  |
| 0 times a week | -.874 | | .364 | -.044 | -1.588, -.16 | -2.401 | **.016** |
| 1 - 2 times a week | -.107 | | .098 | -.02 | -.299, .085 | -1.095 | **.**274 |
| Perceived stress | .052 | | .016 | .087 | .021, 0.83 | 3.298 | **.001** |
| Loneliness | -.125 | | .02 | -.147 | -.165, -.086 | -6.184 | **<.001** |
| Social support | -.052 | | .012 | -.097 | -.076, -.029 | -4.372 | **<.001** |
| Self-efficacy | .031 | | .009 | .071 | .012, .049 | 3.264 | **.001** |
| PHQ-9 | .027 | | .009 | .076 | .009, .045 | 2.885 | **.004** |
| *R*² (*R*² adjusted) | .064 (.06) | |  |  |  |  |  |
| *F* | 15.601 | |  |  |  |  |  |
| *P* | **< .001** | |  |  |  |  |  |
|  |  |  |  |  |  |  |  |

^a^ Marital status dichotomised into “single” and “in a relationship”

^b^ Residential status dichotomised into “alone” and “not alone”

^c^ Being parent dichotomised into “being parent” and “not being parent”

^d^ direct contact with the categories 0, 1-2 and ≥ 3 times a week

^e^ indirect contact with the categories 0, 1-2 and ≥ 3 times a week

Bold font indicates statistical significance, p < .05.

**Table B, supplementary material.** Linear regression analysis for predictors of Bulimia nervosa severity Index, BN-TSI (SEED)

|  |  | Total Sample (*n* = 3,378) | | | | | |
| --- | --- | --- | --- | --- | --- | --- | --- |
| Variable | Unstan-dardized B | | SE B | Standar-dized  *Β* | 95% Confidence Interval (CI) | *t* | *p* |
| Marital status^a^ | .012 | | .015 | .014 | -.017, .042 | .806 | .42 |
| Residential status^b^ | .031 | | .017 | .028 | -.003, .066 | 1.801 | .072 |
| Being parent | .072 | | .031 | .037 | .012, .132 | 2.362 | **.018** |
| Direct contact^d^ |  | |  |  |  |  |  |
| 0 times a week | .014 | | .021 | .015 | -.027, .055 | .65 | .516 |
| 1 - 2 times a week | .013 | | .021 | .015 | -.028, .055 | .622 | .534 |
| Indirect contact^e^ |  | |  |  |  |  |  |
| 0 times a week | -.003 | | .062 | -.001 | -.124, .118 | -.045 | .964 |
| 1 - 2 times a week | -.022 | | .019 | -.018 | -.06, .015 | -1.182 | .237 |
| Perceived stress | .01 | | .003 | .072 | .004, .016 | 3.253 | **.001** |
| Loneliness | -.004 | | .004 | -.019 | -.012, .004 | -.956 | .337 |
| Social support | .002 | | .002 | .017 | -.002, .006 | .921 | .357 |
| Self-efficacy | -.005 | | .002 | -.05 | -.009, -.001 | -2.717 | **.007** |
| PHQ-9 | .032 | | .002 | .390 | .028, .035 | 17.532 | **<.001** |
| *R*² (*R*² adjusted) | .206 (.203) | |  |  |  |  |  |
| *F* | 72.783 | |  |  |  |  |  |
| *P* | **<.001** | |  |  |  |  |  |
|  |  |  |  |  |  |  |  |

^a^ Marital status dichotomised into “single” and “in a relationship”

^b^ Residential status dichotomised into “alone” and “not alone”

^c^ Being parent dichotomised into “being parent” and “not being parent”

^d^ direct contact with the categories 0, 1-2 and ≥ 3 times a week

^e^ indirect contact with the categories 0, 1-2 and ≥ 3 times a week

Bold font indicates statistical significance, p < .05.
